# Long-term Care of Living Kidney Donors Needs a Better Model of Healthcare Delivery

**DOI:** 10.1177/15269248231189879

**Published:** 2023-07-20

**Authors:** Katya Loban, Jorane-Tiana Robert, Ahsan Alam, Shaifali Sandal

**Affiliations:** 1 Division of Nephrology, Department of Medicine, 5620 McGill University Health Centre, Montreal, Canada; 2 Research Institute of the 5620 McGill University Health Centre, Metabolic Disorders and Complications (MeDiC) Program, Montreal, Canada; 3 Division of Experimental Medicine, Department of Medicine, 5620 McGill University Health Centre, Montreal, Canada

**Keywords:** living donors, long-term care, primary care, tertiary care

## Abstract

Every year, over 30,000 healthy individuals globally donate a kidney to a patient with kidney failure. These living kidney donors are at higher risk of some medical complications post-donation when compared with matched controls. Although the absolute risk of these complications is low, appropriate long-term care is essential to allow early detection and timely interventions. Some transplant centers follow living donors long-term, but many recommend that donors regularly see a primary care practitioner post-donation. However, primary care is currently not integrated with transplant centers, and the two often work in silos with little to no channels of communication with each other. As this model of care is suboptimal, existing evidence suggests that post-donation care and follow-up are inadequate. We argue for an integrated model of living donor care with stronger continuity and coordination between primary care and transplant centers that are developed with the input of all relevant stakeholders.

## Introduction

Patients with kidney failure can obtain a kidney from a deceased donor or a living kidney donor (LKD). There is a concerted effort from multiple organizations to increase living donation. This is because patient and graft outcomes of living donor kidney transplantation are superior to deceased donor kidney transplantation.^1^ According to data from the United States, the adjusted 5-year kidney graft survival for living donor transplants from 2015 was 87.1%, compared with 75.6% for deceased donor transplant recipients; the adjusted 5-year patient survival was 87.1% and 76.9%, respectively.^1^ There is a finite number of deceased donors per year, and a discrepancy between the supply and the demand for transplanted organs is a chronic global issue that was exacerbated by the COVID-19 pandemic.^2^ Living donation can narrow this gap. Lastly, some countries lack the infrastructure to run a deceased donor program and living donation is the only way to obtain a transplant.

Living donors are healthy individuals who make this informed, educated, and voluntary donation. According to the Global Observatory on Donation and Transplantation, over 30,000 healthy individuals donate a kidney to a patient with kidney failure annually. They are generally identified and solicited by the patient, referred to as direct LKDs. However, they can be non-directed where they donate without knowing the recipient.

Living kidney donation is not without risks and proper care and follow-up post-donation is required. Donors are screened by a transplant program and undergo a medical, surgical, and psychological evaluation to determine their candidacy prior to donation. After kidney donation, some transplant programs will follow these donors. In 2013, the Organ Procurement and Transplantation Network (OPTN)/United Network for Organ Sharing (UNOS) mandated that transplant centers collect data on LKDs at 6 months, 1 year, and 2 years post-donation with specific thresholds for the proportion of complete follow-up.^3^ Long-term care is generally deferred to primary care practitioners, such as family doctors. In Canada, we conducted a document review including current recommendations, policies, or procedures in place for LKD follow-up care and noted them to be inconsistent (**Table 1**). Some programs/organizations mandate that donors have a family doctor prior to being evaluated for donation, to ensure that they have one post-donation. Primary care is currently not integrated with transplant centers, and the two often work in silos with poor channels of communication with each other. We have highlighted issues with current practices surrounding long-term LKD follow-up and care and argue for an integrated model of care delivery.

**Table 1.** Current Recommendations, Policies, or Procedures in Place for Living Kidney Donor Follow-Up Care with a Family Doctor by Various Organizations in Canada.

Province	Organization	Policies/procedures in place	Illustrative quotes (from websites)
Alberta	Government of Alberta (My health)	Pre-donation care with family doctor, post-donation care may be implied	–
	Transplant program 1^a^	Only donors with a family doctor can proceed with the evaluation	–
	Transplant program 2^a^	No official mandate/policy but patients are encouraged to get a family doctor post-donation	–
British Columbia	BC Renal	No mention	–
	BC Transplant	Implied, reference to some pre-donation testing with family doctor	–
	Transplant program 1^a^	No official documentation but family doctor follow-up is recommended post-donation and not required pre-donation	–
	Transplant program 2^a^	No official mandate/policy but patients are encouraged to get a family doctor pre-donation	–
Manitoba	ODO	Strong recommendation to follow-up with family doctors	Follow-up care: Donors need to maintain a healthy active lifestyle and get regular follow-up with a family doctor once every year or two
	Transplant program^a^	Having a family doctor pre-donation is a requirement	–
Ontario	ODO	No mention in the referral form	–
	Regional renal network	No mention, other than for donors residing outside of Canada	A letter to your family doctor that includes a list of tests you will need to have.
	Transplant program 1^a^	Only donors with a family doctor can proceed with the evaluation	Your family doctor will receive a letter with some recommendations about your ongoing care…. The yearly follow-up can be managed by your family physician. It is essential that you receive life-long follow-up care with blood pressure checks and urine and blood samples to maintain long-term health. It is the responsibility of each living donor to ensure an annual checkup is done through the family doctor or with the donor nephrologist.
	Transplant program 2	Strong recommendation to follow-up with family doctors	Long-Term Follow-Up. Donors need to be seen on a regular basis by their family doctor for a blood pressure reading and laboratory tests to monitor kidney function. The Living Donor Team will send out requisitions for medical testing to the donor and family doctor, and request the results be returned to the Living Donor Program for review. […] Members of the Living Donor Team will be available for any follow-up that may be required.
	Transplant program 3	Recommendation to follow-up with family doctors	For the rest of your life. […] Once a year, see your family doctor to check your kidney function, blood pressure, blood sugar and cholesterol.
	Transplant program 4	Recommendation to follow-up with family doctors	Life-long considerations. After testing at 6 months, the living donor program recommends that you see your family physician annually for the rest of your life. The reason for this is that if you did develop any sign of kidney disease or high blood pressure you should be properly assessed and treated in order to prevent kidney failure. Annual testing would consist of measurement of blood pressure, blood creatinine, and urinalysis. […]Family Doctor. Following your 3-month checkup appointment, you will be discharged back to the care of your family physician. It is suggested that you see your family doctor for a yearly blood pressure check, serum (blood) creatinine level, and a urinalysis
	Transplant program 5	Implied that some pre-donation testing with family doctor	–
	Transplant program 6^a^	Implied that some pre-donation testing with family doctor and transplant program follows many donors	–
Atlantic provinces	Multi-Organ Transplant Program (MOTP)	A mandate to have a family doctor pre-donation	Donors must have a family doctor
Quebec	Government of Quebec	Implied that follow-up is with family doctors	Medical follow-up after the donation. This assessment can be carried out by your family doctor provided that the results are communicated to the living donation team [Translated from French].
	Fondation du rein	No mention	–
	Transplant program 1^a^	No official documentation but family doctor follow-up is recommended post-donation	–
	Transplant program 2^a^	No official documentation but having family doctor is recommended pre-donation	–
	Transplant program 3	NA	–
	Transplant program 4	NA	–
	Transplant program 5	A video online that does not clearly indicate need to follow-up with family doctor	–
Saskatchewan	Transplant Program	NA	–

Abbreviations: NA, not available; ODO, organ donation organization.

Only those ≥18 years of age are eligible for donation. Thus, policies at pediatric transplant programs are not reviewed.

^a^ When confirmed with personnel at the living donor program.

## Clinical Relevancy to Practice

Due to the concerted efforts of multiple researchers, a plethora of literature has emerged over the past decade analyzing the long-term risks of living kidney donation.^4–7^ Donors are at a higher risk of kidney disease, hypertension, and pregnancy-related complications. A systematic review and meta-analysis that included over 100,000 LKDs reported an 8.8 times higher risk for end-stage renal failure when compared with matched controls.^5^ They were also at a 19% higher risk of hypertension.^7^ Women donors were at higher risk for preeclampsia, with an estimated risk of 5.9 events per 100 pregnancies, compared with 3.1 events in control participants.^5^ Of note, while the relative risk for these complications was higher, the absolute risk was generally low. For most donors, the absolute risk of end-stage renal failure was <1%, and risk prediction tools that used demographic and health factors, and innovations in genetic risk markers were improving risk stratification among LKDs.^4^

With emerging evidence, the risk assessment is dynamic and informed consent is an essential component of the donor evaluation. Transplant programs are privy to this literature and routinely integrate recent findings to educate donors regarding risks in the process of donor work-up. If prior LKDs, especially those who donated years ago when these risk assessments were unclear, are aware of this emerging evidence is unknown. Thus, appropriate evidence-informed long-term donor care is a priority. Early detection of complications and referral to the transplant center can allow timely interventions to slow the progression of the disease and the development of other cardiovascular manifestations and improve the long-term outcomes of donors.

## Practice Issue

Despite this, most donors do not receive optimal follow-up even in a country with universal healthcare such as Canada. Donor autonomy has prohibited widespread mandates to have a family doctor. An environmental scan of Canadian living donor programs in 2004 reported that only 25% of transplant programs usually or always provided follow-up beyond the first year of surgery.^8^ Among 534 LKDs in Alberta between 2002 and 2014, over a median follow-up of 7 years, only 25% had complete follow-up care, defined as a physician visit, and serum creatinine and albuminuria measurement in each year of available follow-up.^9^ In the United States, only 43% of centers met the OPTN/UNOS-required 6-month, 1-year, and 2-year follow-up thresholds for LKDs who donated in 2013.^3^ How this care has evolved in light of emerging evidence of long-term risk to living donors is unclear.

Since 2010, there are over 400,000 known LKDs globally as per the Global Observatory on Donation and Transplantation whose care needs are largely unexplored. These data do not include some countries and data related to organ trafficking. Exploring donor perspectives regarding their care needs, increasing access to care via health insurance, and addressing other modifiable risk factors leading to poor follow-up are needed. We raise a practice issue from the system-level perspective. Given that the current model of care delivery is inadequate, how should we deliver optimal care to living donors? Should we strengthen efforts to engage primary care practitioners and then defer all long-term care to them? Should transplant programs take this responsibility and follow all LKDs indefinitely?

Comprehensive, responsive, high-quality primary healthcare, contributes to improved population health outcomes, reduces inequities, improves patient experience and satisfaction, lowers health system costs, and creates more robust healthcare systems.^10^ Long-term follow-up generally entails regular laboratory investigations, blood pressure checks, physical examination, and assessment of mental health. Given these functions and the specific requirements regarding ongoing psychosocial care, the needs of donors may be best addressed by primary care practitioners. In Canada, primary care pivots around family doctors and we studied the family medicine curriculum across Canada's 17 medical schools; none integrate education on living donor care in their curriculum. Primary care practitioners may not be privy to the emerging evidence on long-term risks of living donation or may not be appropriately informed of these risks. There is a dearth of evidence exploring their comfort, attitudes, and knowledge on living donation. Also, in many countries, LKDs do not have regular access to a family doctor as they may lack health insurance.

There are limitations to deferring donor care completely to transplant centers as well. Given their subspecialized training, transplant professionals may lack the expertise to provide ongoing primary care, especially pertaining to care outside of nephrology. Most programs lack the infrastructure needed to provide long-term care. In addition, ongoing care offered by the transplant teams will cost publicly and privately funded healthcare systems more than if it were assumed by primary care practitioners. Moreover, some donors donate in a different state/province/country, which may contribute to non-adherence with follow-ups.

## Implication and Summary

We believe it is important to strike a delicate balance between the needs of living donors and the capabilities of the system. Reviewing donor health data on a regular basis may provide opportunities for improvements in the care delivery and several efforts are ongoing in North America and elsewhere to optimize data collection in national registries. Increasing efforts to improve long-term LKD care requires better care coordination, buy-in from primary care practitioners, commitment from appropriate transplant organizations, and careful training, education, and engagement of all involved.

**Figure 1.**
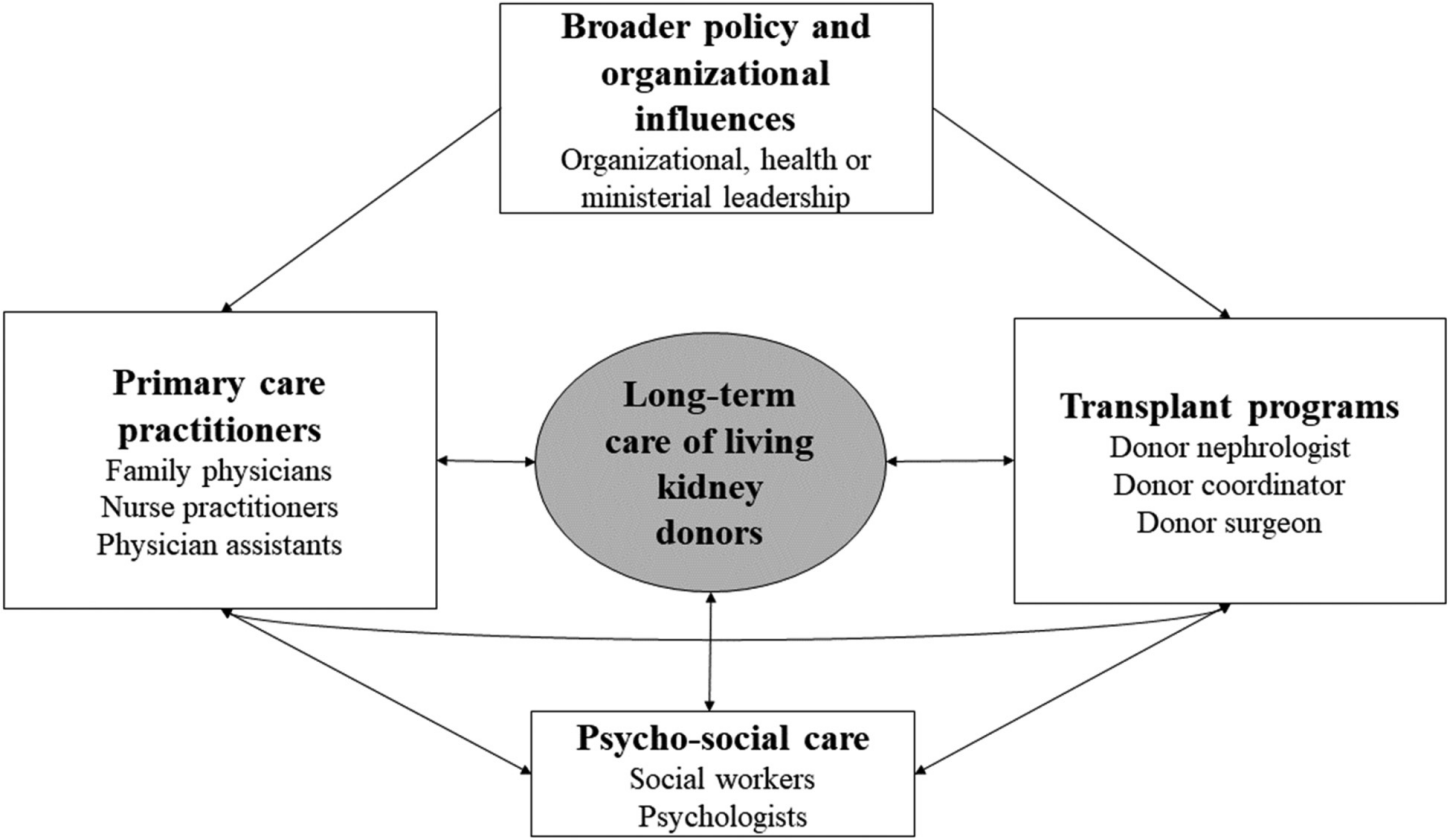
Proposed Model for Integrated Living Donor Care Delivery.

We recommend exploring an integrated model of care with stronger continuity of care between primary and tertiary teams that facilitate the appropriate delivery of healthcare services and overcome fragmentation between providers. This model should include coordination of care that extends beyond the one episode of donation and that embraces the concept of integration across the life course. It should engage all stakeholders, improve modes of communication, and ideally identify opportunities for virtual engagement. The input of relevant stakeholders as shown in **Figure 1**, is needed to develop such a comprehensive and robust model of care and address barriers related to implementation in each region thereafter. This may identify opportunities to strengthen registry data collection as well.

Living donors help save the lives of patients with kidney failure and help to decrease healthcare spending on treatments such as dialysis. Currently, their care is informed by suboptimal care models and does not appropriately engage all stakeholders. We recommend exploring better models of healthcare delivery with transplant programs at the epicenter of this effort and accounting for donors’ perspectives. It is the moral and social responsibility of the entire healthcare system to ensure that living donors receive safe, up-to-date, and high-quality care over their lifetime.
